# Correlation between Epsilon Wave and Late Potentials in Arrhythmogenic Right Ventricular Cardiomyopathy—Do Late Potentials Define the Epsilon Wave?

**DOI:** 10.3390/jcm13175038

**Published:** 2024-08-25

**Authors:** Urszula Skrzypczyńska-Banasik, Olgierd Woźniak, Ilona Kowalik, Aneta Fronczak-Jakubczyk, Karolina Borowiec, Piotr Hoffman, Elżbieta Katarzyna Biernacka

**Affiliations:** Cardinal Wyszynski National Institute of Cardiology, Alpejska 42, 04-628 Warsaw, Poland; uskrzypczynska@ikard.pl (U.S.-B.);

**Keywords:** epsilon wave, late ventricular potentials, arrhythmogenic right ventricular cardiomyopathy, atrial septum defect, Ebstein’s anomaly

## Abstract

**Introduction**: Arrhythmogenic right ventricular cardiomyopathy (ARVC) is a genetic disorder characterised by progressive fibrosis predominantly of the right ventricular (RV) myocardium, resulting in life-threatening arrhythmias and heart failure. The diagnosis is challenging due to a wide spectrum of clinical symptoms. The important role of ECG was covered in the current diagnostic criteria. The role of the epsilon wave (EW) is still under discussion. **Aim**: The aim of the study was to examine a potential association between the EW and late ventricular potentials (LPs) in ARVC patients (pts). The correlation between RV dilatation or dysfunction and LPs/EW was also analysed. **Methods**: The ARVC group consisted of 81 pts (53 men, aged 20–78 years) fulfilling 2010 International Task Force Criteria. 12-lead ECG, LPs, Holter, and ECHO were performed in all pts. The presence of EW was analysed in ECG by 3 investigators. LPs were detected by signal-averaged ECG (SAECG). SAECG was considered positive for LPs when at least two of the three following criteria were met: (1) the filtered QRS duration (fQRS) ≥ 114 msec; (2) the duration of the final QRS fragment in which low-amplitude signals lower than 40 μV are recorded (LAS-40 > 38 msec); and (3) the root mean square amplitude of the last 40 milliseconds of the fQRS complex (RMS-40 < 20 μV). The results were compared with a reference group consisting of 53 patients with RV damage in the course of atrial septum defect (ASD) or Ebstein’s Anomaly (EA). Results: In the ARVC group, a significant relationship was observed between the occurrence of EW and the presence of LPs. EW was more common in the LP+ than in the LP- patients (48.1% vs. 6.9%, *p* < 0001; OR 12.5; 95% CI [2.691–58.063]). In ARVC pts, RVOT > 36 mm, RVIT > 41 mm, and RV S’ < 9 cm/s were observed significantly more often in the LPs+ than in the LPs− group (OR [95% CI]: 8.3 [2.9–1.5], 6.4 [2.2–19.0] and 3.6 [1.1–12.2], respectively). In the ARVC group, any of fQRS > 114 ms, LAS > 38 ms, and RMS < 20 μV were significantly more frequent in EW+ pts. In multivariate analysis, the independent factors of the EW were LAS-40 and RV S’. In the LPs− subgroup, RVOT > 36 mm was more frequent in ASD/EA than in ARVC (70.4% vs. 25%, *p* = 0.002). Similarly, in the LPs− subgroup, RVIT > 41 mm was encountered more frequently in ASD/EA than in ARVC (85.2% vs. 48.3%, *p* = 0.004). **Conclusions**: In ARVC, there is an association between EW and LPs, with both probably resulting from the same process of fibrofatty substitution of the RV myocardium. Although RV dilatation is common in ASD and EA, it does not correlate with LPs.

## 1. Introduction

Arrhythmogenic right ventricular cardiomyopathy (ARVC) is a genetically determined, structural heart disease characterised by the occurrence of ventricular arrhythmias. Necrosis of myocardial fibres of the right ventricular wall and their replacement by fibro-fatty tissue are typical pathophysiological processes of this disease [1]. However, left-dominant or biventricular forms are increasingly recognised [2,3,4,5]. The expression of symptoms is variable (from asymptomatic forms to sudden cardiac death), which makes early diagnosis significantly difficult. In 1994, the first criteria for the diagnosis of ARVC were developed. They included structural, histological, electrocardiographic, arrhythmic, and familial features [6]. The epsilon wave (EW) was the major ARVC diagnostic criterion, whereas late potentials (LPs) were among the minor criteria. Since then, the diagnostic role of EW and LPs remains under debate [7]. In the document proposed in 2020 (“the Padua criteria”), the registration of the epsilon wave in the right precordial leads has become a minor ARVC diagnostic criterion, whereas ventricular late potentials were not included in the electrocardiographic criteria [8].

An epsilon wave is a reproducible low-amplitude signal, occurring at the end of the QRS complex and before the T wave, usually in right precordial leads V1–V3. The presence of an epsilon wave indicates prolonged ventricular depolarisation, resulting from the fibro-fatty infiltration of the myocardium. In severe forms of ARVC with extensive damage to the right ventricle, even multiple epsilon waves may be observed [9]. On standard ECG, an epsilon wave is seen in 15–30% of patients with ARVC. The incidence increases with the use of modified right-sided leads (37%) or bipolar Fontaine leads (57%). An appropriate ECG recording frequency also increases the sensitivity of epsilon wave detection [10,11].

Late potentials are low-amplitude signals at the end of the QRS complex. They are recorded in 50–94% of ARVC patients and correlate with the degree of right ventricle dilatation and systolic dysfunction and also with the occurrence of ventricular tachycardia (VT) [12]. It is known that extensive fibrosis of the myocardium leads to non-homogeneous electrical conductivity, which creates the substrate for the development of ventricular tachycardia (VT) in the re-entry mechanism. LPs as low-amplitude signal waveforms are generally invisible on standard ECG. Therefore, the best method to enhance their registration is SAECG, which is a high-resolution electrocardiography method. Late potentials are detected by removing interference and averaging a large number of high-gain recordings [13,14].

The study aimed to examine a possible association between the epsilon wave and late potentials, which supposedly share the same mechanism. Moreover, the correlation between both phenomena and right ventricle dilatation and dysfunction was investigated. The study group involved ARVC patients, while the control group were patients with congenital heart diseases affecting the right ventricle.

## 2. Methodology

The study was retrospective, and recruitment began in 2017. The 2010 International Task Force Criteria for defining ARVC were followed. Informed consent was obtained from all of the patients. The study was approved by the local ethics committee.

Study population:

1. Study group: ARVC—81 patients with arrhythmogenic right ventricular cardiomyopathy diagnosed on the basis of the International Task Force 2010 Criteria. Late potentials were not taken into account in the diagnosis. This group included 28 women and 53 men, aged 20–78 years.
2. Reference group (with two subgroups):
  - Ebstein’s Anomaly (EA)—24 patients, including 13 women and 11 men, aged 23–55 years.
  - Atrial septal defect (ASD)—29 patients with atrial septal defect scheduled for closure or after closure in adulthood. There were 21 women and 8 men in this group, aged 22–80 years.

None of the patients in both groups had experienced acute coronary syndrome or had a history of myocarditis. Twenty-three (28.3%) patients from the ARVC group had an overt biventricular disease, and none were diagnosed with non-dilated left ventricular cardiomyopathy. Significant tricuspid valve regurgitation was found in 12 ARVC patients (14.8%) and in 15 ASD/EA patients (28.3%).

We compared the ARVC group with the EA and ASD groups due to the similar right ventricle heart failure mechanism, despite demographic differences. Each of these diseases presents RV dilatation with or without systolic dysfunction and low pulmonary pressure. In the terminal stage of ASD with severe shunting, pulmonary hypertension develops. However, if pulmonary hypertension does not occur, there is also low-pressure damage to the right ventricle. None of the ASD patients presented in our study were diagnosed with pulmonary hypertension.

The characteristics of the study groups are presented in Table 1 with mean ±SD or median and quartiles displayed. The left ventricular involvement was defined as LVEF < 55%.

The studied groups differ statistically significantly in terms of gender (ARVC and ASD/EA), age (ASD and EA), and the results of echocardiographic measurements (RVIT, TAPSE, RV S’, LVEF) both before and after age and gender correction. (Table S3).

**Figure 1:**
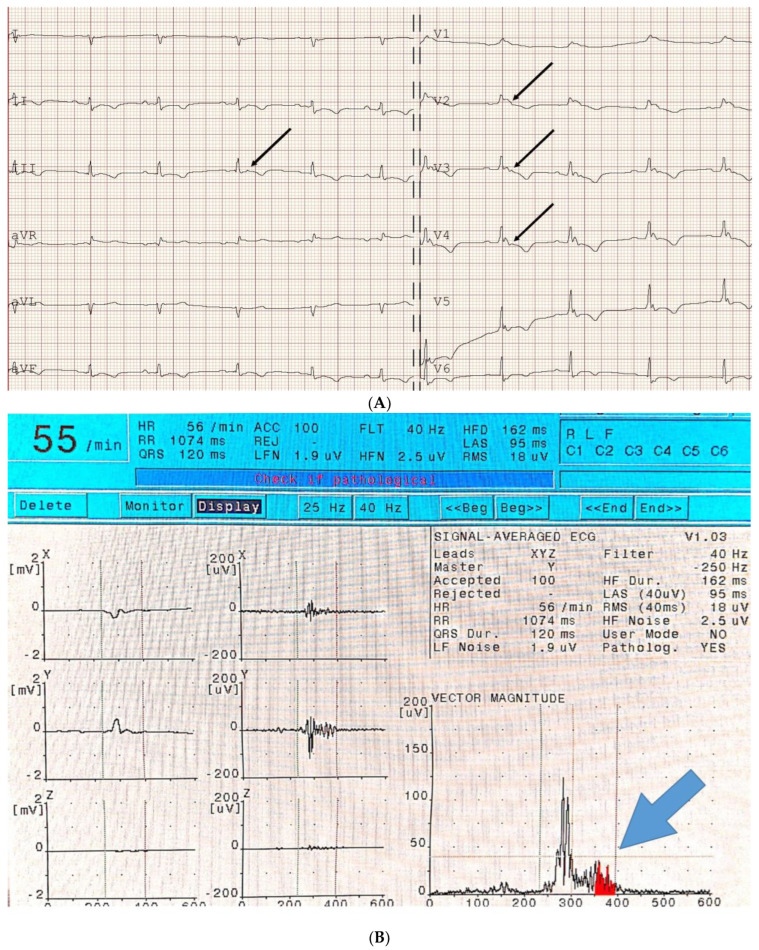
Registration of (**A**) standard 12-lead ECG and (**B**) SAECG in an ARVC (EW+, LP+) patient. The arrows indicate epsilon waves (**A**) and late potentials (**B**).

Each study participant underwent a 12-lead ECG and recording of late ventricular potentials (Figure 1). The latest echocardiographic and Holter ECG recordings were retrospectively analysed. The Epsilon wave, defined as a high-frequency knot occurring at the end of the QRS complex and before the T wave in any lead, was assessed independently by three investigators, including one expert in the field. All doubts regarding the diagnosis of the Epsilon wave were discussed together to establish a common opinion.

SAECG recording was performed using a Schiller AT-10 Plus machine. It was assumed that meeting at least two of the following three criteria allows for the presence of late potentials: (1) fQRS ≥ 114 msec, (2) LAS-40 > 38 msec, and (3) RMS-40 < 20 μV. Echocardiography was analysed with a particular emphasis on the following parameters: the right ventricular outflow tract (RVOT) dimension in the parasternal long-axis view, the dimension of the right ventricular inflow tract (RVIT) in the apical four-chamber projection, and parameters of right ventricular systolic function—the amplitude of the tricuspid annular plane systolic excursion (TAPSE) towards the apex and the systolic (S’) velocity of the tricuspid annular movement assessed by tissue Doppler echocardiography (RV S’).

Then, it was examined whether there was a correlation between the occurrence of late potentials and the epsilon wave or abnormal echocardiographic parameters of the right ventricle.

Additionally, it was assessed whether negative T waves were present in leads V1-V3 of the ECG and whether arrhythmic events occurred.

### Statistical Analysis

All results for categorical variables are presented as counts and percentages and for numerical variables as mean ± SD or median and quartiles (Q1:25th–Q2:75th percentiles). The normality of the distributions of continuous variables was verified using the Shapiro-Wilk test. The chi-square independence or Fisher exact test was used for the comparison of binary variables and Cochran-Mantel-Haenszel general association statistics for nominal data above two categories. The homogeneity of odds ratios was verified by the Breslow-Day test. The significance of differences between paired categorical variables was verified with the Bowker’s symmetry test. The Kappa agreement coefficient was calculated. The differences between numerical variables were tested by the Student’s *t*-test and, in the case of skewed distribution, the Mann-Whitney test. Adjustment of statistics for heterogeneous variables was performed using a general regression model (continuous dependent variable with a normal distribution), robust regression (continuous dependent variable with a skewed distribution), or logistic regression (binary dependent variable), introducing significantly different explanatory variables into the models. Prediction of right ventricular parameters was performed using a general linear regression model and robust regression. The multivariable model includes the variables EW, LP, negative T, age, and gender and uses a backward selection procedure with the criterion of remaining the variable in the model *p* < 0.05. In order to identify independent factors of EW, multivariable analysis was performed using the logistic regression method with backward selection of variables. The input model included variables differentiating EW in univariate analyses. All hypotheses were two-tailed with 0.05 type I error. All statistical analyses were performed using SAS statistical software, version 9.4 (SAS Institute, Cary, NC, USA).

## 3. Results

In the entire group, the presence of late ventricular potentials was detected in 78 patients (58.2%). This included 52 individuals with arrhythmogenic right ventricular cardiomyopathy (64.2% of the subgroup) and 26 with ASD/EA (49.1% of the subgroup) (Table 2).

The epsilon wave was recorded in 31 patients (23.1%), statistically more often in the ARVC group (27 patients, which constituted 33.3% of the subgroup, *p* < 0.001) (Table 2).

In the ARVC group statistically more frequently occurred negative T waves in the precordial leads (53 patients, 65.4%, *p* = 0.002), non-sustained ventricular tachycardias (49 patients, 60.5%, *p* < 0.001), sudden cardiac arrest (9 patients, 11.1%, *p* = 0.012), and RF ablation of ventricular arrhythmias (30 patients, 37.0%, *p* < 0.001).

In the entire group, increased right ventricular outflow tract dimension (RVOT > 36 mm) was found in 81 patients (63.3%), statistically more often in the ASD/EA subgroup (*p* = 0.032). In both subgroups, there were no statistically significant differences in the incidence of increased right ventricular inflow tract dimensions (RVIT > 41 mm), right ventricular end-diastolic area (RVEDa > 22 cm^2^), or right ventricular systolic dysfunction assessed by the following parameters: TAPSE < 16 mm and RV S’ < 9 cm/s (Table 2).

**Figure 2:**
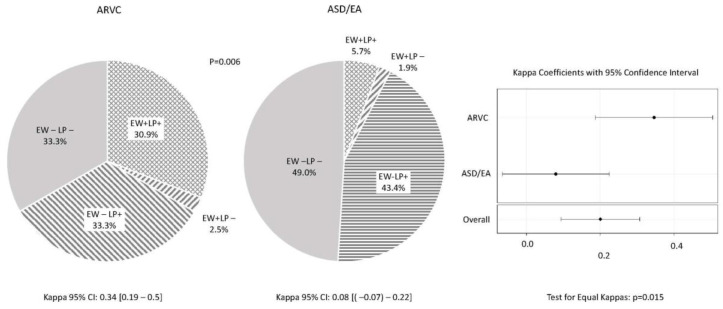
Prevalence of EW and its association with LPs in ARVC and ASD/EA.

In the AVRC group (Figure 2), the presence of an epsilon wave or late potentials was detected in 66.7% of patients, and both features were present in almost 1/3 of them (30.9%). Moreover, LPs occurred significantly more often in the absence of EW than vice versa (33.3 vs. 2.5%, *p* < 0.001). In the control group, however, the simultaneous occurrence of the epsilon wave and late potentials was observed only in 3 (5.7%) patients. Similarly to ARVC, LPs occurred significantly more often in the absence of EW than vice versa (43.4 vs. 1.9%, *p* < 0.001).

The study of the relationship between the frequency of the epsilon wave and late potentials in the study and control groups (Figure 2) showed a significantly different distribution of results in both groups (df = 3, *p* = 0.006), resulting mainly from the significantly more frequent occurrence of both the EW and LPs in the ARVC group (30.9%) than ASD + EA (5.7%), *p* < 0.001. In both groups, the results of both tests were significantly different (CMH test: *p* = 0.006, test for equal Kappas: *p* = 0.015).

In the entire group, we found 5 EW(+) patients who did not meet late potential criteria—3 in the ARVC group and 2 in the ASD/EA group. Each of the two patients in ASD/EA met only one SAECG criterion (fQRS > 114 ms and RMS-40 < 20 μV). We assume the reason was the high noise that prevented the registration of LPs.

**Figure 3:**
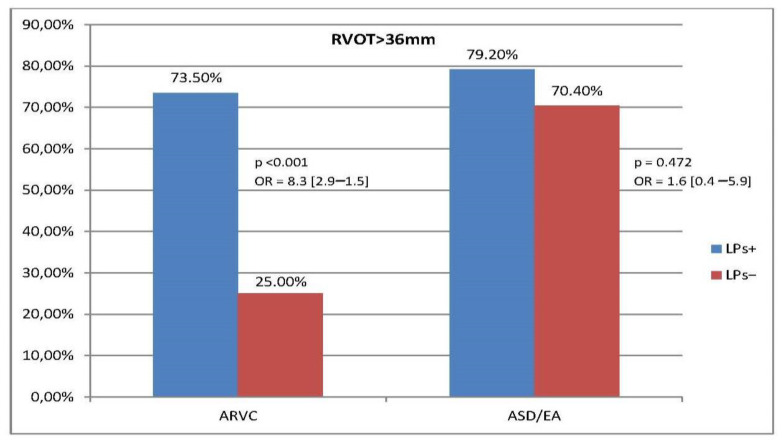
Prevalence of RVOT > 36 mm and association between RVOT diameter and LPs in ARVC and ASD/EA (homogeneity of OR: *p* = 0.049).

**Figure 4:**
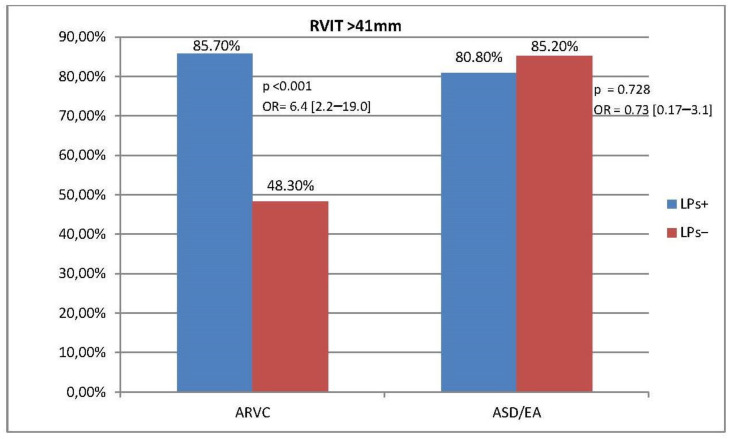
Prevalence of RVIT > 41 mm and association between RVIT diameter and LPs in ARVC and ASD/EA (homogeneity of OR: *p* = 0.015).

**Figure 5:**
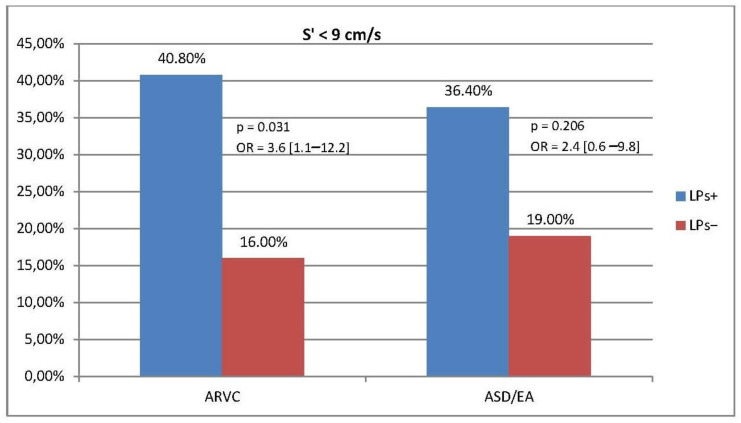
Prevalence of RV S’ < 9 cm/s and association between RV S’ and LPs in ARVC and ASD/EA groups (homogeneity of OR: *p* = 0.049).

In ARVC, a relationship was also found between the presence of late potentials and increased dimensions of the RV inflow tract (RVIT) and right ventricular outflow tract (RVOT) and a reduced S’RV wave value. RVOT > 36 mm, RVIT > 41 mm, and RV S’ < 9 cm/s were observed significantly more often in the LPs+ group than in the LPs− group (OR [95% CI]: 8.3 [2.9–1.5], 6.4 [2.2–19.0], and 3.6 [1.1–12.2], respectively). In the reference group, we did not observe a correlation between LPs and EW as well as LPs and increased RVOT, RVIT, or reduced S’ value. (Figure 3, Figure 4 and Figure 5, Table S1).

**Figure 6:**
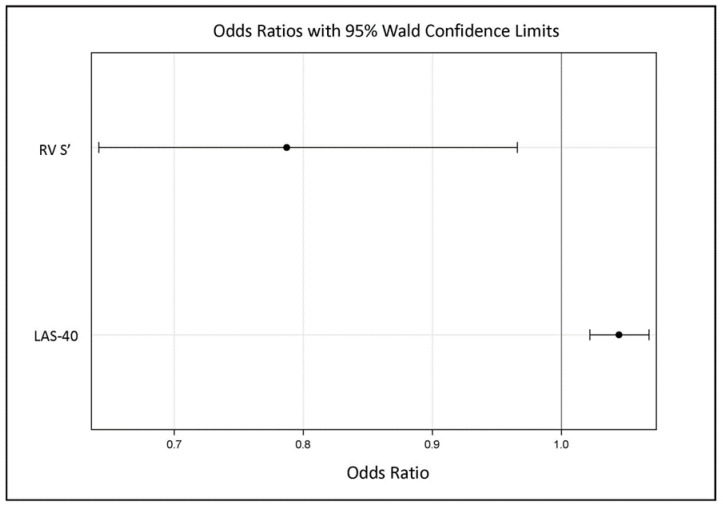
Multivariable logistic regression results for the occurrence of EW.

**Figure 7:**
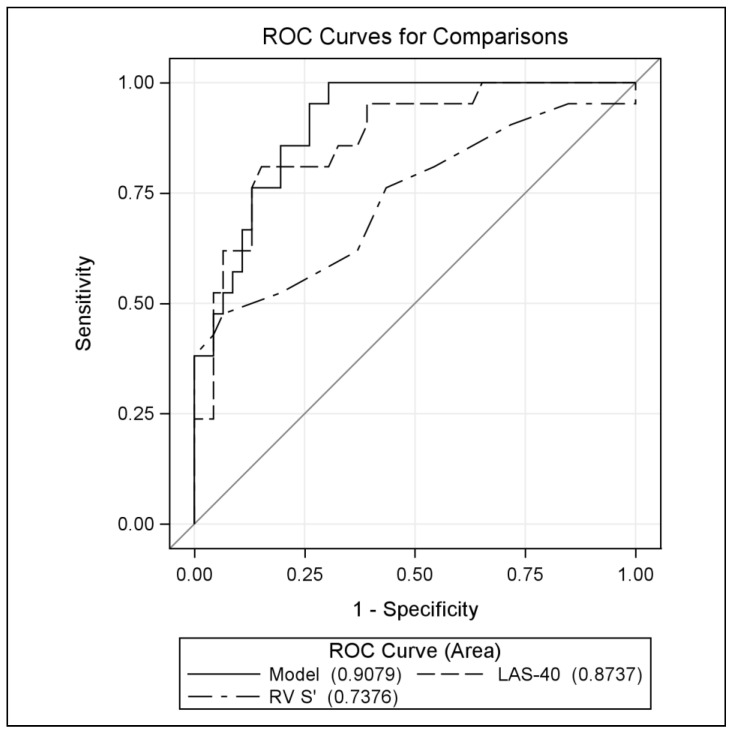
Comparison of ROC curves diagnosing the usefulness of explanatory variables in the model defining the occurrence of EW (dependent variable).

In the ARVC group, any of the three parameters fQRS, LAS-40, and RMS-40 were significantly more common in EW+ patients (Table 3), but in the multivariable analysis, independent factors of EW were LAS-40 (OR [95% CI]: 1.045 [1.022–1.068], *p* < 0.001) and RV S’ (OR [95% CI]: 0.787 [0.641–0.966], *p* = 0.022). The goodness of fit of the model assessed by the area under the ROC curve is: AUC [95% CI]: 0.908 [0.841–0.974] (Figure 6 and Figure 7, Table S2).

In the LPs− subgroup, RVOT >36 mm was more common in ASD/EA than in ARVC (70.4% vs. 25%, *p* = 0.002). Similarly, in the LPs− subgroup RVIT > 41 mm occurred more often in ASD/EA than in ARVC (85.2% vs. 48.3%, *p* = 0.004).

In multivariable analysis, RV dimensions in the ARVC group were related to EW, LPs, and partially to negative T waves. RV function was related to EW, negative T waves, and gender. It was not possible to demonstrate the influence of the association of TAPSE and RV S’ with LPs. Age was an insignificant factor (Table 4).

In the ASD/EA group, RV dimensions and function were associated with negative T waves. Age and gender were insignificant, whereas EW was associated only with RVOT (Table 4).

Greater coefficients of determination for the RVOT, RVIT, and RVEDa models suggest stronger relationships for the ARVC group than for the ASD + EA group.

## 4. Discussion

Arrhythmogenic right ventricular cardiomyopathy is a genetically determined disease that is characterised by the occurrence of ventricular arrhythmias and the risk of sudden cardiac death, especially in young patients and athletes. More than half of SCD/SCA cases did not report previous symptoms or had a known family history of ARVC before their arrest [15]. This makes early diagnosis challenging. The role of epsilon wave and late ventricular potentials was downgraded in the current diagnostic criteria. This reflects the fact that interpretation of the ECG in terms of the presence of an epsilon wave is difficult, and inconsistencies are observed even among experts [16].

The present study indicates that both the epsilon wave and ventricular late potentials may result from the same process of replacement of the myocardium by fibrous tissue. The epsilon wave is not pathogenic for ARVC and may be the electrocardiographic equivalent of prominent late potentials. We suggest that because EW correlates best with duration of the low-amplitude signals (LAS-40), it is therefore an expression of prolonged terminal activation delay (TAD). In ARVC, the epsilon wave and late potentials correlate with right ventricular enlargement and the degree of its dysfunction. This correlation was not observed in ASD/EA despite the frequent occurrence of RV damage in these diseases. This is probably due to the lower degree of RV fibrosis in ASD/EA, but objective quantitative analysis of the RV LGE amount by MRI is difficult due to the small thickness of the right ventricle. In studies in which cardiac MRI was used to assess the left ventricle (ARVC-LV), its involvement in the disease process was reported in 52.4–67% [4,5]. Compared to dilated cardiomyopathy, patients with ARVC-LV had a higher rate of LV LGE (24.6% vs. 13.1%, *p* < 0.01) [4].

Data in the literature regarding myocardial fibrosis in ASD/AE are sparse. In the case of ASD, the process of biatrial fibrosis is primarily emphasised [17]. Fibrous changes in Ebstein’s Anomaly assessed by magnetic resonance imaging in the form of LGE were reported in 16–48.6% of cases [18,19,20], of which 32.4% in the right atrium, 32.4% in the atrialised right ventricle, and 5.4% in the functional right ventricle [18]. There are no quantitative data comparing right ventricular fibrosis processes in ARVC vs. ASD/EA.

The current 2023 ESC Guidelines for the management of cardiomyopathies suggest epsilon waves and SAECG should be used for diagnostic purposes with caution [21]. This study provides a new approach to the utility of EW and SAECG as hallmarks of the histopathological process underlying ARVC that is not observed in other diseases involving RV damage. Both EW and LPs are low-amplitude abnormalities of the final phase of myocardial deporalisation. The reason for their formation is the histological process underlying the disease, leading to dispersion and prolongation of myocardial depolarisation. Both EW and LP recordings depend on the ECG frequency.

In our opinion, the diagnostic significance of LPs as an expression of prolonged depolarisation is underestimated. Although EW is very specific, it is not very sensitive. Moreover, interobserver variability in determining the epsilon wave may lead to a false positive or false negative diagnosis of ARVC. Any doubts in identifying epsilon waves should be resolved by an expert. Registration of LPs by SACG is a more sensitive test.

## 5. Limitations of the Study

The main limitation of the study is the lack of assessment of the correlation of EW and SAECG with the extent of right ventricular fibrosis in magnetic resonance imaging. MRI was not analysed because of difficulties in assessing the presence of fibrous tissue in a thin-walled RV, but above all, it is challenging to quantify it in a reproducible manner. Secondly, many of the patients in our department present advanced or even end-stage disease and already have an implantable cardioverter-defibrillator. In patients with ICD, artefacts do not allow for an accurate and reliable assessment of tissue structure. Other methods, such as high density voltage mapping of RV or myocardial biopsies, are invasive methods. Such testing may only be performed when clinically indicated. Myocardial biopsy may not reveal abnormalities due to the patchy pattern of cardiac fibrosis.

The relationship between LP and EW with RV fibrosis is hypothetical but confirmed by knowledge about the predominant fibrosis in ARVC. This may be a suggestion for further research using new imaging methods.

## 6. Conclusions

In ARVC, late potentials are frequent and correlate with RV dimentions and function. In contrast, late potentials in congenital diseases with RV enlargement (ASD and EA) are observed less frequently and have no correlation with the RV dysfunction. Moreover, in ARVC, there is a relationship between late potentials and the epsilon wave, both of which likely result from the same fibrofatty infiltration of the right ventricular myocardium.

## Figures and Tables

**Table 1:** Study group characteristics. RVOT—right ventricular outflow tract; RVIT—right ventricular inflow tract; RVEDa—right ventricular end-diastolic area; TAPSE—tricuspid annular plane systolic excursion; RV S’—right ventricular systolic excursion velocity; LVEF—left ventricular ejection fraction; LV involvement—left ventricular involvement; number of patients with LVEF < 55%.

	ARVC	ASD + EA	*p*	ASD	EA	*p*
Gender (female)	28 (34.6%)	34 (64.1%)	<0.001	21 (72.4%)	13 (54.2%)	0.168
Mean Age	47.0 ± 15.8	44.5 ± 14.7	0.362	49.4 ± 13.2	38.5 ± 14.5	0.006
RVOT mm	40.4 ± 10.5	44.2 ± 11.7	0.054	40.5 ± 8.9	49.1 ± 13.3	0.013
RVIT mm	47.9 ± 9.2	51.8 ± 11.0	0.030	47.3 ± 8.5	57.2 ± 11.5	<0.001
RVEDa cm^2^	33 (26–42)	29 (24.5–38.5)	0.196	27 (25–30)	35.0 (24–54)	0.060
TAPSE mm	18.6 ± 5.7	20.2 ± 7.8	0.260	22.8 ± 7.2	16.1 ± 7.0	0.010
RV S’cm/s	10.1 ± 3.6	11.6 ± 4.1	0.054	13.3 ± 3.8	9.9 ± 3.7	0.004
LV involvement	23 (28.4%)	3 (5.7%)	0.001	1 (3.4%)	2 (8.3%)	0.584
LVEF %	60 (50–65)	60 (57–85)	0.011	65 (60–65)	59 (55–60)	<0.001

**Table 2:** Echocardiographic and ECG characteristics of the study groups; RVOT—right ventricle outflow tract, RVIT—right ventricle inflow tract, SCA—sudden cardiac arrest, RVA—right ventricle end-diastolic area, TAPSE—tricuspid annular plane systolic excursion, RV S’—right ventricular systolic excursion velocity.

	Total N = 134	ARVC N = 81	ASDE/EA N = 53	*p*	Adjusted for *	
					OR [95% CI]	*p*
Echocardiography						
RVOT > 36 mm *	81 (63.3%)	43 (55.8%)	38 (74.5%)	0.032	0.40 [0.18–0.89]	0.025
RVIT > 41 mm *	100 (76.3%)	56 (71.8%)	44 (83.0%)	0.138	0.42 [0.17–1.05]	0.065
RVEDa > 22 cm^2^ *	101 (87.1%)	58 (90.6%)	43 (82.7%)	0.205	1.54 [0.49–4.88]	0.462
TAPSE < 16 mm *	33 (30%)	23 (31.1%)	10 (27.8%)	0.723	1.12 [0.45–2.79]	0.803
RV S’ < 9 cm/s *	36 (30.8%)	24 (32.4%)	12 (27.9%)	0.609	1.08 [0.46–2.55]	0.863
ECG						
Fullfield 2 criteria of LPs	78 (58.2%)	52 (64.2%)	26 (49.1%)	0.082	3.1 [1.2– 8.1]	0.024
Epsilon wave	31 (23.1%)	27 (33.3%)	4 (7.5%)	<0.001	15.7 [3.2–77.6]	<0.001
Negative T waves (ECG)	73 (54.5%)	53 (65.4%)	20 (37.7%)	0.002	4.9 [1.7–14.6]	0.004

* echocardiography: adjusted for age and gender, ECG: adjusted for age, gender, and echocardiography.

**Table 3:** Comparison of EW+ and EW− patients. OR—odds ratio, CI—confidence interval.

ARVC Group	EW+ n = 27	EW− n = 54	OR [95% CI]	*p*—Wald
Age	50.4 ± 17.5	45.3 ± 14.8	1.021 [0.991–1.053]	0.173
Female	10 (37.0%)	18 (33.3%)	1.176 [0.448–3.086]	0.741
ECG examination				
Negative T-waves	20 (74.1%)	33 (61.1%)	1.8 [0.7–5.0]	0.247
Fullfield 2 criteria of LPS	25 (92.6%)	27 (50%)	12.5 [2.691–58.063]	0.001
HF QRS duration	145.5 ± 30.8	105.3 ± 27.6	1.042 [1.023–1.061]	<0.001
HF_QRS ≥ 114 ms	22 (81.5%)	15 (27.8%)	11.4 [3.7–35.7]	<0.001
LAS-40	95 (69–116)	38 (29–56)	1.041 [1.022–1.060]	<0.001
LAS-40 ≥ 38 ms	25 (92.6%)	28 (51.8%)	11.6 [2.5–53.9]	<0.001
RMS-40	8 (6–14)	18 (8–28)	0.924 [0.875–0.976]	0.004
RMS-40 < 20µV	25 (92.6%)	28 (51.8%)	9.3 [2.0–43.2]	0.004
Delta LAS/RMS	86 (56–114)	20 [(−2)–48]	1.031 [1.017–1.046]	<0.001
Echocardiographic examination				
RVOT	48.5 ± 10.4	36.2 ± 7.7	1.158 [1.080–1.242]	<0001
RVIT	54.8 ± 7.8	44.6 ± 8.0	1.161 [1.078–1.251]	<0.001
RVA	41 (28–45)	31 (25–35)	1.092 [1.027–1.162]	0.005
TAPSE	16.2 ± 6.4	19.7 ± 5.0	0.889 [0.808–0.979]	0.017

**Table 4:** Prediction of right ventricular parameters and function based on ECG parameters, age, and gender in the ARVC and ASD + EA groups. (Multivariable models include the variables EW, LP, negative T, age, and gender and use a backward selection procedure with the criterion of remaining the variable in the model *p* < 0.05. Dependent values: RVOT, RVIT, RVEDa, TAPSE, RV S’. R^2^-Coefficient of determination).

	RVOT		RVIT		RVEDa		TAPSE		RV S’	
	β ± SE	*p*	β ± SE	*p*	β ± SE	*p*	β ± SE	*p*	β ± SE	*p*
ARVC group										
EW	8.9 ± 2.2	<0.001	7.2 ± 1.9	<0.001	4.9 ± 2.5	0.049	−2.7 ± 1.2	0.030	−3.1 ± 0.8	<0.001
LP	7.7 ± 2.1	<0.001	6.2 ± 1.9	0.001	6.6 ± 2.8	0.017	>0.05		>0.05	
Negative T	>0.05		3.7 ± 1.7	0.035	6.2 ± 2.4	0.011	−5.3 ± 1.3	<0.001	−1.8 ± 0.8	0.034
Age	>0.05		>0.05		>0.05		>0.05		>0.05	
Male	>0.05		>0.05		>0.05		−3.6 ± 1.2	0.005	−2.1 ± 0.8	0.012
R^2^	0.415		0.413		0.291		0.289		0.268	
ASD + EA group										
	RVOT		RVIT		RVEDa		TAPSE		RV S’	
	β ± SE	*p*	β ± SE	*p*	β ± SE	*p*	β ± SE	*p*	β ± SE	*p*
EW	12.7 ±5.4	0.025	>0.05		>0.05		>0.05		>0.05	
LP	>0.05		>0.05		>0.05		>0.05		>0.05	
Negative T	8.8 ± 3.0	0.005	9.9 ± 2.8	0.001	12.2 ± 3.7	0.001	−8.3 ± 2.3	0.001	−4.1 ± 1.0	<0.001
Age	>0.05		>0.05		>0.05		>0.05		>0.05	
Male	>0.05		>0.05		>0.05		>0.05		>0.05	
R^2^	0.271		0.192		0.154		0.271		254	

## Data Availability

The data presented in this study are available on request from the corresponding author due to privacy and legal reasons.

## Supplementary Materials

The following supporting information can be downloaded at: https://www.mdpi.com/article/10.3390/jcm13175038/s1, Table S1: Comparison of LP+ and LP− patients. Table S2: Comparison of EW+ and EW- patients. Table S3: Study group characteristics
