# Supplementary material for: Correlation between Epsilon Wave and Late Potentials in Arrhythmogenic Right Ventricular Cardiomyopathy—Do Late Potentials Define the Epsilon Wave?
Source: J Clin Med. 2024 Aug 25;13(17):5038. doi: 10.3390/jcm13175038 (PMC11396235; doi:10.3390/jcm13175038)
Supplement: Supplementary file 1 [file jcm-13-05038-s001.zip › jcm-3121178-supplementary.pdf]

	ARVC				ASD/EA				Homog.	Common OR
	LPs+<br>N=52	LPs-<br>N=29	OR	P	LPs+<br>N=26	LPs-<br>N=27	OR	p		
Epsilon wave	25 (48,1%)	2 (6,9%)	**12,5 [2,7-58,1]**	**<0,001**	3 (11,5%)	1 (3.7%)	3,4 [0,3-34,9]	0,351	0,344	8,9 [2,5-31,3]
Negative T waves (ECG)	37 (71,1%)	16 (55,2%)	2,0 [0,8-5,2]	0,147	11 (42,3%)	9 (33,3%)	1,5 [0,5– 4,5]	0,500	0,676	1,8 [0,9-3,6]
RVOT>36	36 (73,5%)	7 (25,0%)	**8,3 [2,9 – 1,5]**	**<0,001**	19 (79,2%)	19 (70,4%)	1,6 [0,4-5,8]	0,472	**0,0497**	Heterogenous
RVIT>41	42 (85,7%)	14 (48,3%)	**6,4 [2,2 -19,0]**	**<0,001**	21 (80,8%)	23 (85,2%)	0,73 [0,17-3,1]	0,728	**0,015**	Heterogenous
RVA>22	42 (95,5%)	16 (80,0%)	5,2 [0,9-31,5]	0,071	20 (80,0)	23 (85,2)	0,70 [0,16-3,0]	0,722	0,075	1,5 [0,5-4,5]
TAPSE <16	16 (34,8%)	7 (25,0%)	1,6 [0,6-4,6]	0,378	5 (33,3%)	5 (23,8%)	1,6 [0,4-7,0]	0,709	1,00	1,6 [0,7-3,8]
S’<9	20 (40,8%)	4 (16,0%)	**3,6 [1,1-12,2]**	**0,031**	8 (36,4%)	4 (19,0%)	2,4 [0,6 – 9,8]	0,206	0,601	3,1 [1,2 – 7,6]

Table. S1. Comparison of LPs+ and LPs- patients

	**ARVC**				**ASD/EA**				Homog.	Common OR
	Epsilon+ N=27	Epsilon- N=54	OR [95% CI]	P	Epsilon+ N=4	Epsilon- N=49	OR [95% CI]	p		
LPs	25 (92,6%)	27 (50,0%)	**12,5 [2,7–58,1]**	**<0,001**	3 (75,0%)	23 (46,9%)	3,4 [0,3 – 34,9]	0,351	0,344	8,9 [2,5-31,3]
HF_QRS≥114	22 (81,5%)	15 (27,8%)	11,4 [3,7-35,7]	**<0,001**	4 (100%)	21 (42,9%)	2,3 [1,7-3,2]*	**0,043**	0,486	13,7[4,4-42.9)
LAS >=38	25 (92,6%)	28 (51,8%)	11,6 [2,5-53,9]	**<0,001**	3 (75,0%)	23 (46,9%)	3,4 [0,3-34,9]	0,351	0,373	8,4 [2,4-29,7]
RMS≤20	25 (92,6%)	31 (57,4%)	9,3 [2,0 – 43,2]	**0,001**	3 (75,0%)	23 (46,9%)	3,4 [0,3-34,9]	0,351	0,471	7,1 [2,0-25,4]

Table. S2. Comparison of EW+ and EW- pts

* relative risk HFQRS≥114

	**ARVC**	**ASD+EA**	**P**	**ASD**	**EA**	**P**
**Adjusted for gender**				**Adjusted for age**		
**RVOT mm**	40.5 [38.0 - 43.1]	44.1 [41.0 – 47.2]	0.089	40.2 [36.0 – 44.5]	49.5 [44.6 – 54.5]	**0.007**
**RVIT mm**	47.6 [45.3 – 49.9]	52.1 [49.3 – 54.9]	**0.016**	46.8 [43.0 – 50.7]	57.7 [53.5 – 62.0]	**<0.001**
**RVEDa cm2**	31.0 [31.0 – 34.9]	32.6 [28.7–32.6]	0.267	27.0 [26.4 –27.6]	33.7 [33.2 – 34.3]	0.027
**TAPSE mm**	18.9 [17.4 – 20.4]	19.9 [17.8 – 22.1]	0.438	22.7 [19.4 – 25.9]	16.4 [12.3 – 20.5]	**0.026**
**RV S'cm/s**	10.3 [9.3 – 11.2]	11.5 [10.3 – 12.6]	0.115	13.6 [11.8 – 15.3]	9.6 [7.9 – 11.3]	**0.004**
**LVEF %**	59.6 [58.8 – 59.6]	60.5 [60.5 – 61.4]	0.203	63.3 [63 – 63.6]	58.2 [57.9– 58.5]	**<0.001**

Table S3. Study groups characteristics
